# Validation of community health worker identification of maternal puerperal sepsis using a clinical diagnostic algorithm in Bangladesh and Pakistan

**DOI:** 10.7189/jogh.11.04039

**Published:** 2021-11-27

**Authors:** Amnesty E LeFevre, Fatima Mir, Dipak K Mitra, Shabina Ariff, Diwakar Mohan, Imran Ahmed, Shazia Sultana, Peter J Winch, Sadia Shakoor, Nicholas E Connor, Mohammad Shahidul Islam, Shams El-Arifeen, MA Quaiyum, Abdullah H Baqui, Michael G Gravett, Mathuram Santosham, Zulfiqar A Bhutta, Anita Zaidi, Samir K Saha, Saifuddin Ahmed, Sajid Soofi, Linda A Bartlett

**Affiliations:** 1 School of Public Health and Family Medicine, University of Cape Town, Cape Town, Western Cape, South Africa; 2 Department of International Health, Johns Hopkins Bloomberg School of Public Health, Baltimore, Maryland, USA; 3 Department of Paediatrics and Child Health, The Aga Khan University, Karachi, Pakistan; 4 Department of Public Health, North South University, Dhaka, Bangladesh; 5 The Child Health Research Foundation, Department of Microbiology, Dhaka Shishu Hospital, Dhaka, Bangladesh; 6 Department of Clinical Research, London School of Hygiene & Tropical Medicine, London, United Kingdom; 7 Centre for Child and Adolescent Health, icddr,b, Dhaka, Bangladesh; 8 University of Washington, Departments of Obstetrics & Gynecology and of Global Health, Seattle, Washington, USA; 9 Centre of Excellence in Women & Child Health, The Aga Khan University, Karachi, Pakistan; 10 Centre for Global Child Health, Hospital for Sick Children, Toronto, Canada; 11 Department of Population, Family and Reproductive Health, Johns Hopkins Bloomberg School of Public Health, Baltimore, Maryland, USA

## Abstract

**Background:**

Puerperal sepsis (PP sepsis) is a leading cause of maternal mortality globally. The majority of maternal sepsis cases and deaths occur at home and remain undiagnosed and under-reported. In this paper, we present findings from a nested case-control study in Bangladesh and Pakistan which sought to assess the validity of community health worker (CHW) identification of PP sepsis using a clinical diagnostic algorithm with physician assessment and classification used as the gold standard.

**Methods:**

Up to 300 postpartum women were enrolled in each of the 3 sites 1) Sylhet, Bangladesh (n = 278), 2) Karachi, Pakistan (n = 278) and 3) Matiari, Pakistan (n = 300). Index cases were women with suspected PP Sepsis as diagnosed by CHWs clinical assessment of one or more of the following signs and symptoms: temperature (recorded fever ≥38.1°C, reported history of fever, lower abdominal or pelvic pain, and abnormal or foul-smelling discharge. Each case was matched with 3 control women who were diagnosed by CHWs to have no infection. Cases and controls were assessed by trained physicians using the same algorithm implemented by the CHWs. Using physician assessment as the gold standard, Kappa statistics for reliability and diagnostic validity (sensitivity and specificity) are presented with 95% CI. Sensitivity and specificity were adjusted for verification bias.

**Results:**

The adjusted sensitivity and specificity of CHW identification of PP sepsis across all sites was 82% (Karachi: 78%, Matiari: 78%, Sylhet: 95%) and 90% (Karachi: 95%, Matiari: 85%, Sylhet: 90%) respectively. CHW-Physician agreement was highest for moderate and high fever (range across sites: K = 0.84-0.97) and lowest for lower abdominal pain (K = 0.30-0.34). The clinical signs and symptoms for other conditions were reported infrequently, however, the CHW-physician agreement was high for all symptoms except severe headache/ blurred vision (K = 0.13-0.38) and reported “lower abdominal pain without fever” (K = 0.39-0.57).

**Conclusion:**

In all sites, CHWs with limited training were able to identify signs and symptoms and to classify cases of PP sepsis with high validity. Integrating postpartum infection screening into existing community-based platforms and post-natal visits is a promising strategy to monitor women for PP sepsis - improving delivery of cohesive maternal and child health care in low resource settings.

Globally, an estimated 295 000 maternal deaths occurred in 2017; Southern Asia including Bangladesh and Pakistan accounted for 20% of these deaths [1]. Puerperal sepsis (PP sepsis) is the third most frequent cause of maternal mortality worldwide [2]. In 2013, over 30 000 maternal deaths (10.7%: 95% CI: 5.9-18.6) were attributed to PP sepsis; almost all occurred in low resource settings, with the highest proportion reported in South Asia (13.7%) [2]. Beyond high rates of mortality, morbidity from PP sepsis affects 5% to 10% of pregnant women globally and is associated with severe or disabling complications, including chronic pelvic inflammatory disease and infertility [3]. Further adverse fetal outcomes, including pre-term birth, neonatal septicemia, pneumonia, and a depressed 5-minute Apgar score, may additionally occur as a result of infection transmission to newborns [4-9].

Efforts to reduce the burden of PP sepsis have largely focused on facility-based interventions to prevent infections, and promote early identification and treatment. However, the timing of puerperal sepsis, coupled with high rates of home deliveries and low utilization of postnatal care services [10] result in most sepsis cases and deaths occurring at home and remaining undiagnosed and under-reported [3]. Evidence emerging from Bangladesh [11], India [12-15], Ghana [16,17], Pakistan [18], Nepal [19,20], South Africa [21-23], and Zambia [24,25], suggest that integrated packages of community-based services provided by community-health workers (CHWs) may be an effective strategy for addressing critical gaps in human resources, reducing morbidity for women, mortality and morbidity for newborns, and improving care-related outcomes [26]. In Bangladesh, CHWs equipped with clinical algorithms for assessing newborns, have demonstrated the ability to identify key clinical signs and symptoms of severe illness with a high level of validity as part of routine, population-based household surveillance [27,28]. To date, however, no studies have explored the feasibility and effectiveness of utilizing CHWs for community-based maternal PP sepsis identification and management.

Building upon previous work to detect and manage newborn sepsis at the community level [11,29,30], the *Aetiology of Neonatal Infection in South Asia* (ANISA) was established as a multi-country study to determine the incidence and etiology of community acquired neonatal infections in South Asia [31]. A supplemental study was conducted in three sites to explore three objectives: to describe the incidence and risk factors of PP sepsis; determine the etiology; and evaluate the validity of CHW identification of maternal PP sepsis using a clinical diagnostic algorithm in Bangladesh and Pakistan. In this manuscript, we report findings from the CHW PP Sepsis algorithm validation component.

## METHODS

### Study sites, design and sampling

This nested case-control study was conducted from 2012 to 2014 in the rural sites of Sylhet, Bangladesh and Matiari, Pakistan and the peri-urban site in Karachi, Pakistan, described in detail elsewhere [31,32]. To evaluate the CHW algorithm and its implementation required a ratio of 1 case to 3 controls for a total of 300 women per site, including 75 women suspected by CHWs as having PP sepsis (cases), and 225 healthy women (controls). This sample size is sufficient to assess for sensitivity of 95% and specificity of 85% with 5% and 15% margins of errors, respectively, with 5% Type-I (α) error. Due to the controlled sampling design of the study, the ratio of cases to controls will determine the prevalence of the condition according to the formula n_cases_/ n_controls_ = prevalence/ 1-prevalence. Due to sensitivity and specificity <100%, the final true prevalence in the sample (as determined by gold standard) will be less than the pre-specified 25% (75/300) and the final precision interval will be more than 5% specified.

### Clinical algorithm

**Figure 1:**
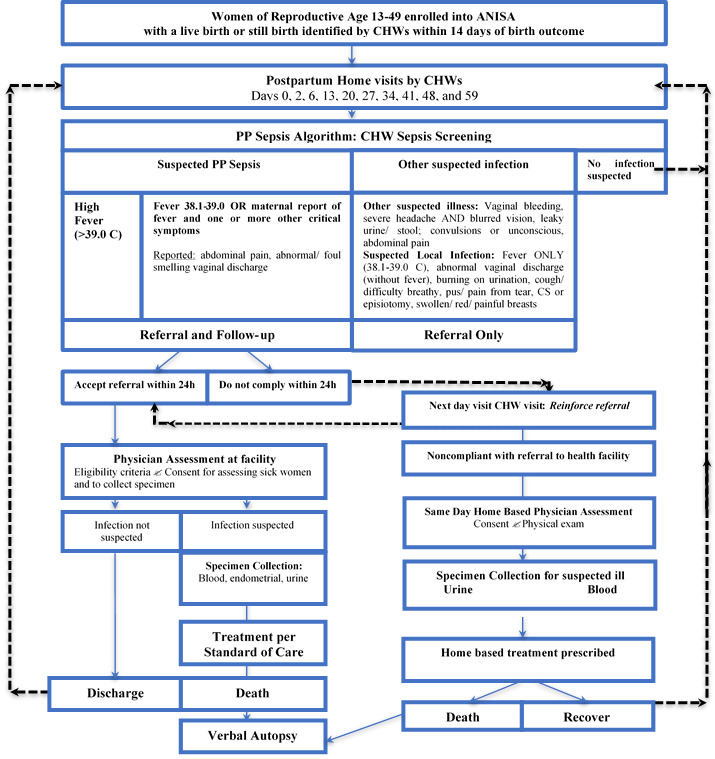
Surveillance system for postpartum maternal infection. In Matiari, women suspected to have sepsis by CHWs were assessed in the home a physician. Confirmed cases were treated in the home with oral antibiotics. Blood and urine were collected in the home. Endometrial specimens were collected in a government health facility the following day.

Formative research determined local knowledge of symptoms and signs of PP sepsis and a systematic literature review was conducted to design a diagnostic algorithm for CHWs to use during ten postpartum home visits [33]. The draft clinical algorithm reviewed by the study Technical Advisory Committee (TAG) [32] sought to facilitate the identification of endometritis – a post-partum infection of the lining of the uterus, which occurs between onset of the rupture of membranes or labour and 42 days postpartum. As defined by the World Health Organization (WHO), endometritis is characterised by fever and one or more of the following symptoms: pelvic pain, abnormal vaginal discharge, abnormal odour of discharge, and delay in the rate of reduction of size of the uterus (<2 cm/ day during the first 8 days) [34]. Consistent with these WHO criteria, presumptive cases of PP sepsis were classified based on i) the CHW’s recorded temperature recorded with thermometer: high fever: >39.0°C, or fever: 38.1°C – 39.0°C); or ii) client verbally reported history of fever in addition to one of the following symptoms, lower abdominal or pelvic pain, and abnormal or foul-smelling discharge (**Table 1**). Following collection of data on signs and symptoms, CHWs were asked to classify illness into one of five categories: suspected sepsis, other suspected illness, suspected local infection, other or no infection (**Figure 1**).

**Table 1:** Simplified algorithm for community health worker identification of postpartum maternal infection

Signs and symptoms screened by community health workers	Classification
*High* fever: temperature 102.4°F (39.1°C) or higher	Suspected puerperal sepsis
Fever: temperature 100.6°F-102.3°F (38.1°C-39.0°C)	Suspected puerperal sepsis if fever present at examination or history of fever AND any other sign or symptom listed is present
History of fever	
Lower abdominal or pelvic pain	
Abnormal or foul-smelling discharge	
Severe vaginal bleeding	Other suspected illness
Severe headache AND blurred vision	
Leaking urine and/or stool	
Convulsions or unconscious	
Lower abdominal pain (without fever)	
Fever only: temperature 100.6°F-102.3°F (38.1°C-39.0°C)	Suspected local infection
History of fever only	
Abnormal or foul-smelling vaginal discharge (without fever)	
Burning on micturition	
Cough or difficulty breathing	
Pus or pain from tear, c- section or episiotomy wound	
Swollen, red, or painful breast	

### Disease surveillance, identification, and management

Across all sites, CHWs register married women of reproductive age (13-49 years), identify pregnancies during bi-monthly surveillance visits, conduct birth preparedness visits at 12-20 weeks and 28–30 weeks of pregnancy and carry out 10 postpartum home visits (Days 0, 6, 13, 20, 27, 34, 41, 48 and 59 postpartum) among live birth newborns [31]. The study enrolled consenting women for maternal infection surveillance with live birth and stillbirth outcomes identified within 14 days of the birth outcome (the main ANISA trial enrolls newborns identified up to 7 days following delivery. Amongst those birth outcomes identified between days 8 and 14, only the mothers are enrolled for maternal infection surveillance). Women with any suspected PP infection were referred to tertiary care facilities in Sylhet and Karachi, and visited in the home by a study physician in Matiari, given its rural location. In Sylhet and Karachi, among suspected cases of PP sepsis that did not comply with referral recommendations, follow up home visits were carried out by CHWs within 24 hours. In Matiari, in the event that the study physician deemed additional referral necessary, women were referred to CIVIL Hospital, a tertiary care teaching hospital in Hyderabad. Among individuals that still refused referral, study physicians performed same day home visits and if the diagnosis was confirmed, prescribed an oral treatment regimen. Antibiotic regimens were informed by both the WHO recommendations and a systematic literature review conducted as part of this study [35]. Women who consented to referral and admission to hospital for treatment were managed according to the local standard of care. In the event of death of a woman, a verbal autopsy was implemented among consenting family members to determine cause of death. Women with symptoms of other suspected illness or local infection were also referred, but not followed up within 24 hours for physician visit or re-referral.

### CHW clinical algorithm validation

For every suspected ill case assessed by CHWs in each site, 3 healthy women were randomly selected as controls who did not have or report any PP sepsis related signs and symptoms, who were +/− 1 day in terms of days postpartum and were from the same administrative area. In Karachi and Sylhet, suspected ill women and selected controls were referred to a site health facility for assessment by a study physician and in cases of non-adherence to referral, assessed in the home. In Matiari, study physicians conducted assessments in the home given the rural nature of study setting. In all sites, the study physicians’ diagnostic practice was standardized using training materials about PP sepsis based on WHO’s Manual of Complications in Pregnancy and Childbirth (MCPC) diagnostic criteria (fever, chills, lower abdominal pain, purulent, foul smelling lochia, tender uterus, +/− light vaginal bleeding and signs and symptoms of shock) [36]. Study physicians also received remote training by both local and a US-based based obstetrician experienced with the pulmonary biopsy tool (Tao Sampling by Cook Medical Brush) for endometrial biopsy. Biopsy specimens were sent for aerobic and anaerobic culture and molecular detection of bacterial etiologic agents that were previously identified and/or plausible [37].

### Statistical analysis

Data were analyzed in Stata 13 (Stata Corporation, College Station, TX, USA). The unit of analysis were CHW–physician assessment pairs. Associations between physicians and CHWs assessments were examined across sites and overall at two levels: the CHW and physicians classification of maternal infections (using the combination of symptoms and signs, as guided by the algorithm) as either suspected PP sepsis, other suspected illness, or suspected local infection. Physician agreement and sensitivity and specificity of individual signs and symptoms were calculated. To cross-check CHW categorization of suspected PP sepsis we additionally synthesized findings from individual signs and symptoms identified to derive a second estimate of what the anticipated CHW categorization would have been if CHWs correctly identified all the signs. The physicians’ assessment and classification were considered the gold standard for calculating sensitivity and specificity. Sensitivity and specificity were adjusted for verification bias a piori. Kappa statistics (K) were calculated to determine agreement between CHWs and physicians [38]. To adjust kappa coefficients for differences in the prevalence levels of an attribute, as well as random and/or systematic differences between CHW and physician ratings, Prevalence Adjusted Bias Adjusted Kappa (PABAK) scores were derived and are presented in the results as K. Prevalence indices account for differences in the prevalence of an attribute; where the index is high - chance agreement may also be high and correspondingly, the kappa reduced [39]. Similarly, to adjust for differences in extent to which CHWs and physicians disagreed on the proportion of positive or negative cases of infection, a bias index was calculated [39]. Low bias indices denote greater symmetry in CHW-physician disagreement, while high indices reflect asymmetry in CHW-physician disagreement [39]. We present both unadjusted kappa and PABAK coefficients, with the latter shown to demonstrate the likely effects of prevalence and bias alongside the true value of K in the study sites. Finally, positive and negative agreement between CHWs and physicians is shown, reflecting agreement on the presence or absence of an attribute, respectively. Receiver operating characteristic (ROC) curves, which the plot of sensitivity vs 1-specificity, were plotted and the area under the ROC (AUROC) calculated to illustrate how well the CHWs using the algorithm are able to distinguish between true and false classification of women as PP sepsis cases.

### Ethical review

The study was approved by the Institutional Review Board (IRB) at Johns Hopkins, Bloomberg School of Public Health and the Ethical Review Committees at the International Centre for Diarrheal Disease Research, Bangladesh, the Aga Khan University in Pakistan, and the Child Health Research Foundation in Bangladesh.

## RESULTS

Analyses included 878 women across the three study sites. In each site, the study sample included the first 75 cases and 225 controls who completed the interview, were identified within 0-42 days postpartum and assessed by a physician within 2 days of the CHW assessment.

### Detection of signs and symptoms

**Table 2**, **Table 3** and **Table 4** summarize findings on the CHW-Physician agreement on the presence and/or absence of signs and symptoms in the three sites. Of the two clinical signs (high and moderate fever) and three symptoms (history of fever, lower abdominal pain, foul/ abnormal discharge) used to identify suspected PP sepsis, only lower abdominal pain (27%-38%) was observed with modest frequency across all three sites. In Matiari and Karachi, reported history of fever was additionally observed in one-third of women. Recorded fever (high temperature) was only rarely recorded by thermometer at the time of CHW or physician examination, and reported abnormal vaginal discharge were reported in 11% of cases in Matiari, 6% in Karachi and 4% in Sylhet.

**Table 2:** Kappa statistics for agreement between assessments by community health workers and physicians in Sylhet, Bangladesh (n = 278)

	Frequency (%)*	Agreement (%)		Overall agreement (%)	Kappa		SE	*P*-value
**+**	**–**	**Adjusted**	**Unadjusted**
**Suspected puerperal sepsis / Other suspected illness / Suspected local infection**								
**Clinical signs:**								
High fever: temperature 102.4°F (39.1°C) or higher	0.4	100	99	97	0.95	0.22	0.04	<0.01
Fever: Temperature 100.6°F-102.3°F (38.1°C-9.0°C)	0.7	6	96	93	0.86	0.15	0.05	<0.01
**Symptoms:**								
History of fever	13	45	94	87	0.80	0.67	0.06	<0.010
Lower abdominal or pelvic pain	38	19	63	67	0.34	0.37	0.05	<0.01
Abnormal or foul-smelling discharge	4.0	13	95	91	0.83	0.43	0.06	<0.01
**Other conditions**								
**Clinical signs:**								
Fever only: temperature 100.6°F-102.3°F (38.1°C-39.0°C)	0.4	100	100	100	0.99	0.00	0.00	–
**Symptoms:**								
Severe vaginal bleeding	1.4	7	96	92	0.84	0.23	0.06	<0.01
Severe headache and blurred vision	15	5	77	68	0.13	0.00	0.00	<0.01
Leaking urine and/or stool	0.0	–	100	100	–	0.00	0.00	–
Convulsions or unconscious	1.1	–	100	99	0.96	0.00	0.00	0.50
Lower abdominal pain (without fever)	17	–	77	69	0.39	0.35	0.05	<0.01
History of fever only	13	45	94	87	0.80	0.67	0.06	<0.01
Abnormal or foul-smelling vaginal discharge (without fever)	0.4	5	98	95	0.91	0.11	0.06	0.03
Burning on micturition	9.4	10	88	81	0.62	0.39	0.06	<0.01
Cough or difficulty breathing	4.7	6	91	84	0.68	0.28	0.06	<0.01
Pus or pain from tear, c-section or episiotomy wound	2.9	31	98	95	0.91	0.53	0.06	<0.01
Swollen, red, or painful breast	1.4	12	96	92	0.84	0.24	0.05	<0.01

SE – standard error

*Physician and community health worker assessed as positive.

**Table 3:** Kappa statistics for agreement between assessments by community health workers and physicians in Matiari, Pakistan (n=300)

	Frequency (%)*	Agreement (%)		Overall agreement (%)	Kappa		SE	*P*-value
+	**–**	**Adjusted**	**Unadjusted**
**Suspected puerperal sepsis / Other suspected illness/suspected local infection**								
**Clinical signs:**								
High fever: temperature 102.4°F (39.1°C) or higher	0.7	6	98	97	0.94	0.30	0.05	<0.01
Fever: Temperature 100.6°F-102.3°F (38.1°C-39.0°C)	2.0	10	96	92	0.84	0.29	0.06	<0.01
**Symptoms:**								
History of fever	32	83	84	81	0.61	0.60	0.06	<0.01
Lower abdominal or pelvic pain	27	64	68	65	0.30	0.31	0.05	<0.01
Abnormal or foul-smelling discharge	11	100	86	78	0.56	0.36	0.06	<0.01
**Other conditions**								
**Clinical signs:**								
Fever only: temperature 100.6°F-102.3°F (38.1°C-39.0°C)	0.3	–	–	100	–	0.00	0.06	0.52
**Symptoms:**								
Severe vaginal bleeding	1.0	60	93	87	0.75	0.11	0.04	<0.01
Severe headache and blurred vision	35	76	69	69	0.38	0.38	0.06	<0.01
Leaking urine and/or stool	0.7	–	–	99	–	0.00	–	–
Convulsions or unconscious	0.0	–	–	99	–	-0.01	0.05	0.53
Lower abdominal pain (without fever)	7.3	54	87	78	0.57	0.28	0.06	<0.01
History of fever only	32	76	84	78	0.57	0.55	0.06	<0.01
Abnormal or foul-smelling vaginal discharge (without fever)	1.7	34	93	87	0.75	0.14	0.06	<0.01
Burning on micturition	5.3	41	94	89	0.77	0.42	0.06	<0.01
Cough or difficulty breathing	12	100	87	80	0.61	0.43	0.06	<0.01
Pus or pain from tear, c- section or episiotomy wound	4.3	30	97	94	0.87	0.54	0.06	<0.01
Swollen, red, or painful breast	2.3	22	91	85	0.69	0.17	0.05	<0.01

SE – standard error

*Physician and community health worker assessed as positive.

**Table 4:** Kappa statistics for agreement between assessments by community health workers and physicians in Karachi, Pakistan (n = 300)

	Frequency (5)*	Agreement (%)		Overall agreement (%)	Kappa		SE	*P*-value
**+**	**–**	**Adjusted**	**Unadjusted**
**Suspected puerperal sepsis / Other suspected illness / Suspected local infection**								
**Clinical signs:**								
High fever: temperature 102.4°F (39.1°C) or higher	0.7	100	100	99	0.97	0.00	–	–
Fever: Temperature 100.6°F-102.3°F (38.1°C–39.0°C)	0.7	44	99	98	0.97	0.44	0.05	<0.01
**Symptoms:**								
History of fever	29	76	85	81	0.63	0.61	0.06	<0.01
Lower abdominal or pelvic pain	38	69	63	66	0.33	0.32	0.06	<0.01
Abnormal or foul-smelling discharge	6.3	46	91	85	0.70	0.37	0.06	<0.01
**Other conditions**								
**Clinical signs:**								
Fever only [temperature 100.6°F-102.3°F (38.1°C-39.0°C)]	0.0	–	–	–	–	–	–	–
**Symptoms:**								
Severe vaginal bleeding	0.7	15	96	92	0.85	0.11	0.06	0.03
Severe headache and blurred vision	14	45	76	66	0.32	0.25	0.05	0.00
Leaking urine and/or stool	0.7	100	100	99	0.97	0.00	0.00	–
Convulsions or unconscious	0.3	33	99	99	0.97	0.33	0.04	<0.01
Lower abdominal pain (without fever)	12	49	83	75	0.50	0.33	0.06	<0.01
History of fever only	29	76	85	81	0.63	0.61	0.06	<0.01
Abnormal or foul-smelling vaginal discharge (without fever)	1.0	21	96	92	0.85	0.17	0.06	<0.01
Burning on micturition	11	57	90	84	0.68	0.48	0.06	<0.01
Cough or difficulty breathing	0.7	8	90	84	0.80	0.42	0.06	<0.01
Pus or pain from tear, c- section or episiotomy wound	8.3	54	92	86	0.72	0.47	0.06	<0.01
Swollen, red, or painful breast	11	53	88	80	0.61	0.41	0.06	<0.01

SE – standard error

*Physician and community health worker assessed as positive.

Across the three sites, CHW-Physician agreement was highest for moderate and high fever (K>0.84, *P* < 0.001; overall agreement of ≥92%) and lowest for lower abdominal pain (K = 0.30-0.34; *P* < 0.001. While the frequency of reporting of clinical signs and symptoms for other conditions was low, CHW-physician agreement was high for all symptoms but severe headache/ blurred vision (K = 0.13-0.38) and lower abdominal pain without fever (K = 0.39-0.57). Across sites, similar trends were observed, with almost all of the Kappa scores reaching statistically significant levels. While the prevalence of clinical signs and symptoms for the other non-PP sepsis conditions was of low to moderate prevalence, CHW-physician agreement was high for all symptoms except for severe headache/ blurred vision (K = 0.13-0.38, *P* < 0.001) and lower abdominal pain without fever (K = 0.39-0.57, *P* < 0.001). Across sites, similar trends were observed.

**Table 5**, **Table 6** and **Table 7** document data on the frequency and validity of historical factors reported by mothers, as well as the clinical signs and symptoms observed by CHWs. In Sylhet, sensitivity (Se) and specificity (Sp) were very high for recorded high fever (Se = 100%; Sp = 98%) and reported history of fever (Se = 90%; Sp = 90%). The other PP Sepsis signs and symptoms showed low sensitivity (recorded moderate fever (Se = 33%), lower abdominal/pelvic pain (Se = 57%) and abnormal vaginal discharge (Se = 48%)), however, specificity was high (≥88%). For all other signs and symptoms assessed, sensitivity was low ranging from 13% for vaginal bleeding to 57% for lower abdominal pain, while specificity exceeded 87%. In Matiari, among PP sepsis symptoms and symptom assessed, low sensitivity and high specificity were generally observed: high fever (Se = 22%; Sp = 100%), moderate fever (Se = 32%; Sp = 96%), history of fever (Se = 78%; Sp = 83%), lower abdominal pain (Se = 75%; Sp = 59%), and vaginal discharge (Se = 58%; Sp = 82%). The sensitivity for symptoms of other clinical conditions showed a range of very low sensitivity (vaginal discharge with no fever, Se = 23%) to high (severe headache and blurred visions, Se = 71%; history of fever with no other clinical signs or symptoms, Se = 78%). Specificity was high for all symptoms assessed with the exception of severe headache or blurred vision (Sp = 67%). In Karachi, a similar picture emerged. Among PP sepsis symptoms and symptom assessed, low sensitivity and high specificity were generally observed: moderate fever (Se = 33%; Sp = 100%), history of fever (Se = 69%; Sp = 90%), lower abdominal pain (Se = 69%; Sp = 64%), and vaginal discharge (Se = 39%; Sp = 94%). Sensitivity for symptoms of other clinical (non-PP Sepsis) conditions ranged from 13% for severe vaginal bleeding to 71% for severe headache and blurred vision; high specificity was seen throughout.

**Table 5:** Sensitivity and specificity of historical factors reported by mothers and clinic signs and symptoms observed by community health workers across sites in Sylhet, Bangladesh (n = 278)

	TP	TN	FP	FN	Sensitivity			Specificity		
**%**	**95% CI**		**%**	**95% CI**	
**Suspected puerperal sepsis / Other suspected illness / Suspected local infection**										
**Clinical signs:**										
High fever: temperature 102.4oF (39.1°C) or higher	1	270	7	0	100	3	100	98	95	99
Fever: Temperature 100.6°F-102.3°F (38.1°C-39.0°C)	2	257	15	4	33	4	78	95	91	97
**Symptoms:**										
History of fever	37	214	23	4	90	77	97	90	86	94
Lower abdominal or pelvic pain	107	79	11	81	57	50	64	88	79	94
Abnormal or foul-smelling discharge	11	243	12	12	48	27	69	95	92	98
**Other conditions**										
**Clinical signs:**										
Fever only: temperature 100.6°F-102.3°F (38.1°C-39.0°C)	1	277	0	0	–	–	–	–	–	–
**Symptoms:**										
Severe vaginal bleeding	4	252	15	7	36	11	69	94	91	97
Severe headache and blurred vision	41	149	22	66	38	29	48	87	81	92
Leaking urine and/or stool	0	277	1	0	–	–	–	–	–	–
Convulsions or unconscious	3	275	0	0	–	–	–	–	–	–
Lower abdominal pain (without fever)	48	145	9	76	39	30	48	94	89	97
History of fever only	37	214	23	4	90	77	97	90	86	94
Abnormal or foul-smelling vaginal discharge (without fever)	1	264	6	7	13	0	53	98	95	99
Burning on micturition	26	199	12	41	39	27	52	94	90	97
Cough or difficulty breathing	13	220	14	31	30	17	45	94	90	97
Pus or pain from tear, c- section or episiotomy wound	8	257	4	9	47	23	72	99	96	100
Swollen, red, or painful breast	4	252	3	19	17	5	39	99	97	100

**Table 6:** Sensitivity and specificity of historical factors reported by mothers and clinic signs and symptoms observed by community health workers across sites in Matiari, Pakistan (n = 300)

	TP	TN	FP	FN	Sensitivity			Specificity		
**%**	**95% CI**		**%**	**95% CI**	
**Suspected puerperal sepsis / Other suspected illness / Suspected local infection**										
**Clinical signs:**										
High fever: temperature 102.4°F (39.1°C) or higher	2	289	2	7	22	3	60	100	98	100
Fever: Temperature: 100.6°F-102.3°F (38.1°C-39.0°C)	6	270	11	13	32	13	57	96	93	98
**Symptoms:**										
History of fever	95	147	31	27	78	70	85	83	76	88
Lower abdominal or pelvic pain	81	114	78	27	75	66	83	59	52	66
Abnormal or foul-smelling discharge	32	202	43	23	58	44	71	82	77	87
**Other conditions**										
**Clinical signs:**										
Fever only (temperature 100.6°F-102.3°F (38.1°C–9.0°C)]	1	299	0	0	–	–	–	–	–	–
**Symptoms:**										
Severe vaginal bleeding	3	259	35	3	50	12	88	88	84	92
Severe headache and blurred vision	105	102	50	43	71	63	78	67	59	75
Leaking urine and/or stool	2	298	0	0	.	.	.	.	.	.
Convulsions or unconscious	0	296	1	3	0	0	71	100	98	100
Lower abdominal pain (without fever)	22	213	42	23	49	34	64	84	78	88
History of fever only	95	147	31	27	78	70	85	83	76	88
Abnormal or foul-smelling vaginal discharge (without fever)	5	257	21	17	23	8	45	92	89	95
Burning on micturition	16	250	23	11	59	39	78	92	88	95
Cough or difficulty breathing	37	204	34	25	60	46	72	86	81	90
Pus or pain from tear, c- section or episiotomy wound	13	268	8	11	54	33	74	97	94	99
Swollen, red, or painful breast	7	247	37	9	44	20	70	87	83	91

TP – true positive, TN – true negative, FP – false positive, FN – false negative, CI – confidence interval

**Table 7:** Sensitivity and specificity of historical factors reported by mothers and clinic signs and symptoms observed by community health workers across sites in Karachi, Pakistan (n = 300)

	TP	TN	FP	FN	Sensitivity			Specificity		
**%**	**95% CI**		**%**	**95% CI**	
**Suspected puerperal sepsis / Other suspected illness / Suspected local infection**										
**Clinical signs:**										
High fever: temperature 102.4°F (39.1°C) or higher	2	298	0	0	–	–	–	–	–	–
Fever: Temperature 100.6°F-102.3°F (38.1°C-39.0°C)	2	293	1	4	33	4	78	100	98	100
**Symptoms:**										
History of fever	87	157	17	39	69	60	77	90	85	94
Lower abdominal or pelvic pain	113	86	49	52	69	61	76	64	55	72
Abnormal or foul-smelling discharge	19	236	15	30	39	25	54	94	90	97
**Other conditions**										
**Clinical signs:**										
Fever only: temperature 100.6°F-102.3°F (38.1°C-39.0°C)	0	300	0	0	–	–	–	–	–	–
**Symptoms:**										
Severe vaginal bleeding	2	275	9	14	13	2	38	97	94	99
Severe headache and blurred vision	42	157	84	17	71	58	82	65	59	71
Leaking urine and/or stool	2	298	0	0	–	–	–	–	–	–
Convulsions or unconscious	1	295	4	0	100	3	100	99	97	100
Lower abdominal pain (without fever)	36	189	42	33	52	40	64	82	76	87
History of fever only	87	157	17	39	69	60	77	90	85	94
Abnormal or foul-smelling vaginal discharge (without fever)	3	274	9	14	18	4	43	97	94	99
Burning on micturition	32	220	17	31	51	38	64	93	89	96
Cough or difficulty breathing	2	225	28	21	55	40	70	89	84	93
Pus or pain from tear, c- section or episiotomy wound	25	233	10	32	44	31	58	96	93	98
Swollen, red, or painful breast	33	208	19	40	45	34	57	92	87	95

TP – true positive, TN – true negative, FP – false positive, FN – false negative, CI – confidence interval

### CHW classification of illness

**Figure 2:**
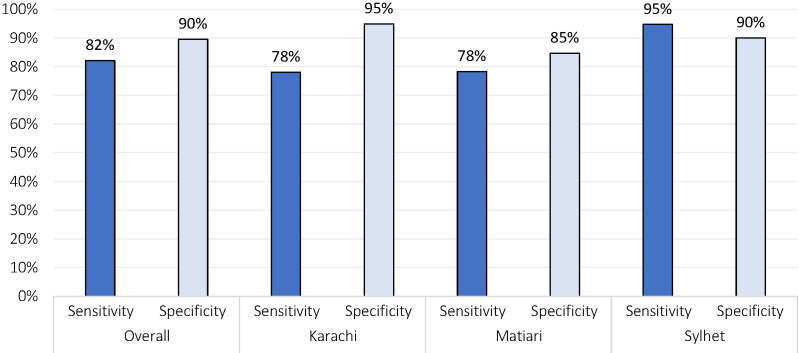
Adjusted sensitivity and specificity for CHW identification of PP sepsis vs Physician assessment across study sites.

Following collection of data on signs and symptoms, CHWs were asked to classify illness into one of five categories: suspected sepsis, other suspected illness, suspected local infection, other or no infection. Sensitivity and specificity analyses were adjusted for verification bias and are presented across sites in **Figure 2**. The combined site sensitivity of CHW’s correct classification of PP sepsis was 82% (range across sites of 78% - 95%) and specificity 90% (range 85%-95%).

**Figure 3:**
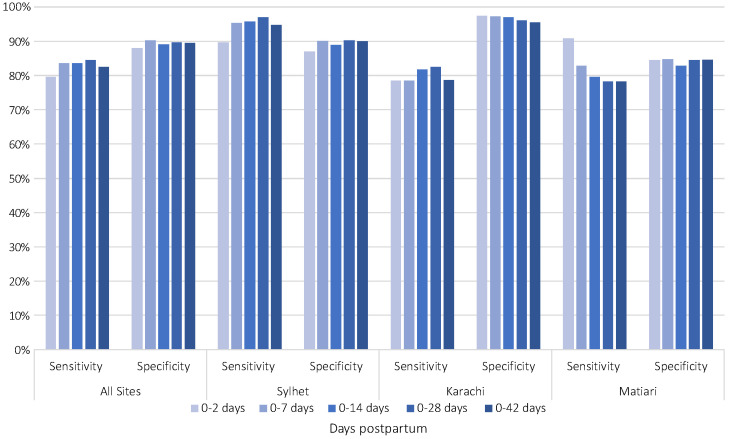
Adjusted sensitivity and specificity by days postpartum across sites.

To explore the validity of the CHW’s application of the algorithm over time, we measured the adjusted sensitivity and specificity of CHW diagnosis of sepsis by groupings of days postpartum in the following increments: 0-2 days, 0-7 days, 0-14 days, 0-28 days and 0-42 days (**Figure 3**). Overall results suggest that the adjusted sensitivity and specificity were similar by day postpartum.

## DISCUSSION

This study sought to assess the validity of CHW identification of maternal puerperal sepsis using a clinical diagnostic algorithm in Bangladesh and Pakistan with physician assessment and classification used as the gold standard. CHWs were able to identify and classify suspected cases of PP sepsis with high sensitivity (82%) and specificity (90%) in all sites. The AUROC measures were also promising: indicating a probability of 89%-97% that CHWs equipped with the algorithm could correctly distinguish between positive and negative cases. The CHW and physician overall agreement was also high for four of five signs and symptoms assessed for PP sepsis: documented high and moderate fever, history of fever and abnormal vaginal discharge, reaching statistical significance in this assessment of CHW: physician agreement. The most sensitive and specific of the signs and symptoms of PP sepsis also included documented high fever and reported history of fever. However, high fever was rarely actually documented via thermometer: only reported history of fever was of moderate prevalence. The other signs – also more prevalent - while specific, were less sensitive. Across all sites, the sensitivity and specificity were similar by days postpartum; indicating that CHWs were able to apply the tool throughout the postpartum window with a high degree of validity.

While not the primary focus of the study, the opportunity to save lives by asking women about other life-threatening or serious complications via the algorithm could not be ignored. Kappa statistics varied by site, but the signs or symptoms with high Kappa’s common to the three sites were severe vaginal bleeding; convulsions or unconsciousness; abnormal or foul vaginal discharge (without fever); and pus or pain from tear, C/S or episiotomy wound (Sylhet and Karachi only) (all adjusted Kappas >0.80). Severe vaginal bleeding and convulsions or altered consciousness are critical signs of late post-partum hemorrhage and severe pre-eclampsia/eclampsia respectively – both serious life-threatening complications requiring urgent referral to specialized obstetric care. Other conditions such as abnormal vaginal discharge (without fever) or cough are less worrisome symptoms but also require non-urgent referral to an appropriate level of care. However, while not consistently prevalent across sites, leaking urine or stool strongly suggests a bladder or rectal vaginal fistula, or severe damage to the pelvic floor – either of which are serious issues that require surgical intervention. And swollen, red or painful breast suggest mastitis, an infection of the breast that can become serious for the mother and contribute to inadequate milk production – impacting the newborn. These require validation in different contexts but show promise as community-based method to identify other post-partum complications.

The strong performance of CHWs across all sites may have been enhanced by the experience of the implementing partners and their well-established CHWs programs. In Sylhet, CHWs have been providing community-based services for nearly two-decades. Assessments dating back to 2005-2006 have shown that CHWs were able to correctly classify very severe disease in newborns with a sensitivity of 91%, specificity of 95%, and kappa of 0.85 (*P* < 0.001) [27]. Successful implementation of the algorithm in addition to other community-based activities ultimately contributed to a 34% reduction in neonatal mortality [11]. CHWs have subsequently been used to provide family planning [40], promote umbilical cord cleansing with chlorhexidine [41], and most recently, to screen and treat maternal genitourinary tract infections with the aim of preventing pre-term births [42]. In Matiari, the well-established national cadre of “Lady Health Workers” have been utilized as the cornerstone of community-based service delivery. For nearly a decade, the Matiari site has sought to test innovations to the standard package of community-based activities, including the use of CHWs to promote antenatal care and maternal health education, use of clean delivery kits, facility births, immediate newborn care, identification of danger signs, and careseeking. Collectively these have activities have been shown to significantly reduce neonatal mortality (RR 0.85, 0.76-0.96; *P* = 0.02) [43]. Among children under 5, CHWs in Matiari have also demonstrated effectiveness in implementing community case management of WHO-defined severe pneumonia; a process requiring intensive screening of children in addition to treatment and follow-up [44]. In Karachi, while CHW programmatic activities are more nascent when compared against the two other sites, teams have been operating for a nearly a decade; providing services to low income residents in peri-urban coastal fishing villages. Proximity to secondary and tertiary care facilities, has meant that much of the programmatic content of services has emphasized rapid identification of illness and referral.

This is one of the first studies to equip CHWs in a low resource setting with a diagnostic tool for real-time postpartum infection screening in the home, and to our knowledge – the only one to date to assess CHW’s ability to use an algorithm to identify post-partum women with prospective PP sepsis. Reports found on other studies to validate alternative strategies to identify maternal morbidity describe different retrospective methods and have demonstrated mixed results. In Benin, midwife-administered questionnaires in home and clinic settings at 6 months postpartum demonstrated poor validity for detecting common postpartum morbidities including anemia (34% sensitivity, 66% specificity), incontinence (5% sensitivity, 98% specificity), UTIs (2% sensitivity, 95% specificity), and prolapse (18% sensitivity, 91% specificity) [45]. In Brazil, efforts to compare maternal recall of complications related to pregnancy and childbirth with medical records similarly suggested that women could not accurately recall the occurrence of obstetric complications, including hemorrhage and infection, although recollection of process indicators like hysterectomy or blood transfusion were much higher. The length of time after delivery for these queries was not specified, but the authors note that increasing length of time from the delivery was associated with poorer recall [46]. Our study assessing CHW algorithm use was administered to post-partum women – asking about their current symptoms – rather than specific complications such as hemorrhage or care interventions and consequently does not have the potential recall bias of other morbidity measurement methods and tools.

Elsewhere in the literature, encouraging evidence on the effectiveness of screening for conditions in the home is emerging. Among newborns, CHWs have been used effectively to screen for fetal alcohol spectrum disorders [47], categorize weight in Uttar Pradesh, India [48] as well as conduct infection screening in several South Asian settings, including Mirzapur [28] and Sylhet [27], Bangladesh; and Gadchiroli, Maharashtra, India [49]. Among children under 5, CHWs have demonstrated effectiveness in screening for neurodevelopmental status [50], as well as pneumonia [44], diarrhea [51], and malaria [52]. In Sylhet, a randomized controlled trial is under way to screen and treat pregnant women between 13 and 19 weeks of gestation for abnormal vaginal flora and urinary tract infections –as a means to prevent pre-term birth [42]. Project activities rely on CHWs to provide routine antenatal and postnatal home-based care in addition to screening and treatment of pregnant women between 13 and 19 weeks of gestation for abnormal vaginal flora and urinary tract infections [42]. As part of postnatal care activities, CHWs assess mothers for vital status, fever, uterine tenderness and symptoms of postpartum hemorrhage [42]. Women with suspected postpartum complications (defined as fever >38.3°C, abdominal/uterine tenderness, self-report of excessive hemorrhage) are identified and referred to health facilities for additional care and treatment [42]. While the project is presently under way, measurement of maternal morbidity including PP sepsis is anticipated to be a key outcome. Beyond this study, no additional studies were identified in the literature which emphasize community-based postpartum infection screening in low resource settings and none were found to emphasize maternal post-partum screening focussed on puerperal sepsis.

As dialogue continues on the optimal scope of work for CHWs [53], efforts are needed to improve integration of care and capitalize on opportunities for treating both mothers and newborns. Study findings here point to the infrequency of signs and symptoms of maternal postpartum infection which could render stand-alone vertical PP sepsis programs cost ineffective. However, post-partum care for mothers and newborns is an area in great need of strengthening [54]. This study, and the ANISA study with its underpinning newborn algorithms may provide an opportunity for generating recommendations on an integrated tool for maternal and newborn infection screening. In settings where CHWs are already in the community and/or home conducting screening for newborns, a more comprehensive approach to service delivery which includes maternal screening is likely to incur minimal incremental costs and concurrently yield improvements in morbidity and mortality. Our findings suggest that CHWs can identify symptoms and signs with very good accuracy as compared to physicians. A community-based screening algorithm should be highly sensitive – so that women suspected of illness can be referred for more expert diagnoses. The most sensitive symptoms and signs were measured fever, and asking about a history of fever, and should be included in other algorithms evaluated or deployed elsewhere. While less sensitive, the limited number of questions needed to ask about the more prevalent signs, the high sensitivity and specificity of CHW overall PP sepsis classification, and the AUROC measures indicate that the spectrum of questions about lower abdominal or pelvic pain and abnormal vaginal discharge also be included in algorithms to optimize correct identification of women who may have PP sepsis and require referral for management. As analyses continue on the risk factors for both maternal and newborn infection, further refinement to the recommended number and timing of home visits may additionally emerge and allow for further programmatic streamlining. While context specific adaptations of the algorithm may be necessary, testing in two settings in two South Asian countries provides a strong foundation for further assessments elsewhere in the region where other cadres of CHWs exist, including in India and sub-Saharan Africa where the majority of maternal deaths occur. While the symptoms and signs of PP sepsis should not differ physiologically between regions, the capabilities of CHWs including their ability to collect clinical data on signs and symptoms may differ.

### Limitations

The validation sub-study was part of a larger study on maternal infection which sought to additionally determine the incidence, risk factors and etiology of maternal infection in South Asia. In an effort to capture risk factors and measure incidence, a larger questionnaire was developed for use by CHWs which included a section specific to the algorithm which summarized signs and symptoms gathered through queries earlier in the instrument. The assessment of the tool was not predictive, ie, do these symptoms identified actually predict impending sepsis. Rather, our analyses falls under the category of criterion validity assessment – determining if the CHW and physician came to the same conclusion using the tool at (approximately) at the same point in time [55]. Future applications of this algorithm are likely to streamline questions which may facilitate implementation, minimize the potential for reporting and classification errors as well as the time required to assess women, and ultimately, improve the validity of the tool. Study implementation occurred in research sites through cadres of CHWs which are likely to differ from government supported frontline health workers deployed as part of national programs. The successful replication of findings from Sylhet, Karachi and Matiari will depend on a number of factors including the underlying capabilities of CHWs, their existing workloads, quality of initial and in-service training, supervisory structures and referral systems. In settings where CHWs have lower levels of literacy and supporting structural inputs are lacking, validity is likely to be lower. Our findings represent the prevalence in the population-based surveillance systems used in this study and may not reflect the risk in the entire country.

## CONCLUSIONS

We endeavored to include mothers in post-partum screening programs that heretofore focused on newborns: assessing the validity of CHW identification of maternal puerperal sepsis using a clinical diagnostic algorithm in Bangladesh and Pakistan with physician assessment and classification used as the gold standard. CHWs with limited training can use a diagnostic algorithm to identify signs and symptoms and classify cases of PP sepsis with high validity. Evaluations of maternal infection providing a forum for emphasizing more integrated care and equal prioritization of mothers in addition to newborns during a period of peak vulnerability. Integrating postpartum infection screening into existing community-based platforms is a promising strategy for improving delivery of integrated maternal and child health care in low resource settings.
